# A New Method of Fixing High-Aspect-Ratio Microstructures by Gel

**DOI:** 10.3390/mi7070115

**Published:** 2016-07-09

**Authors:** Nan Chen, Xiangyu Chen, Penghui Xiong, Shuangyue Hou, Xiaobo Zhang, Ying Xiong, Gang Liu, Yangchao Tian

**Affiliations:** 1 Department of Precision Machinery and Precision Instrumentation, University of Science and Technology of China, Hefei 230026, China; judechen@mail.ustc.edu.cn (N.C.); cxy0910@mail.ustc.edu.cn (X.C.); xph@mail.ustc.edu.cn (P.X.); 2 National Synchrotron Radiation Laboratory, University of Science and Technology of China, Hefei 230029, China; hsy58@mail.ustc.edu.cn (S.H.); zhangxb8@ustc.edu.cn (X.Z.); xywch@ustc.edu.cn (Y.X.)

**Keywords:** gel, fix, high-aspect-ratio microstructures

## Abstract

In the microfabrication processes, it is necessary to examine the quality of the structures to ensure the whole process runs smoothly. However, the examination process of pattern defects is interrupted during the fabrication of high-aspect-ratio microstructures. The inevitable pattern defects arise from capillary forces which occur during the liquid drying process. In this paper, a new method that enables us to fix the microstructures with gel to restrict deformations before the rinsed liquid drying process has been proposed. It is effective to avoid the capillary forces by preventing the formation of the liquid level. The process parameters, types of gel, gel time and observation time were discussed and the flatness and thickness of the gel layer could be controlled. A series of high-aspect-ratio microstructures were fixed in good condition by gel.

## 1. Introduction

The high-aspect-ratio microstructures have some clear advantages and have good performance in optics, the adhesion of antimicrobials, dynamics, catalytic action, etc. As important optical devices for X-ray imaging, the gratings and zone plates, which have a small linewidth and high aspect ratio, are able to obtain high resolution in the imaging process [1,2]. In general, during the complex microfabrication processes, it is necessary to monitor the quality of the structures after each process. Particularly, photoresist microstructures should carefully be examined after photolithography and then the faultless photoresist microstructures would be transferred to the further process, such as electroplate. However, it is difficult to observe the features of high-aspect-ratio microstructures fabricated by lithography.

Many pattern defects are generated due to capillary forces during the liquid drying process [3,4,5]. Capillary forces could be avoided by preventing the direct phase in changing from liquid to gas. Freeze-drying and supercritical drying are techniques that can avoid pattern defects [6,7]. However, the temperature varies greatly in both of them, easily resulting in structure deformations. Additionally, in the supercritical drying process, it is found that carbon dioxide is incorporated into photoresists such as polymethyl methacrylate, permanently changing the geometry of the microstructures or leading to severe damage [8]. Therefore, a tolerant process called “plating always in liquid” is used to avoid pattern collapse during the fabrication of metal high-aspect-ratio microstructures [9]. Once the rinse process is finished, the sample will be immediately immersed into the electroplating solution for plating. The sample is in liquid during the whole process in order to avoid capillary forces. However, it is difficult to monitor the quality of the patterns and form a standard electroplating process when the sample is always in liquid. Therefore, it is necessary to find a universally applicable method which can be used to fix the structures effectively, and then to observe the quality of the patterns.

In this paper, a new method of fixing the high-aspect-ratio microstructures is proposed. The structures can be fixed by gel before the rinsing and liquid drying process. Capillary forces are eliminated by avoiding the formation of the liquid level. Pattern defects will not occur in the whole process, which ensures the test results reflect the real fabrication quality. A method for controlling the flatness and thickness of the gel layer is presented. For a series of high-aspect-ratio structures fixed by gel, clear and stable observations were achieved. The sol-gel fixing technique is not a finishing step and it is a process for temporarily fixing wetted microstructures. After examination of the photoresist microstructures under the microscope, the gel could be readily dissolved in warm water to allow further processing of the sample.

## 2. Materials and Methods

### 2.1. Conventional Defects

**Figure 1:**
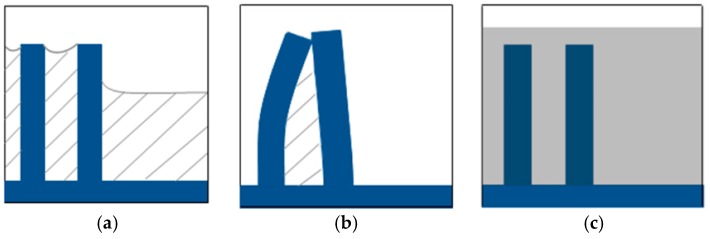
(**a**) Evaporation of liquid during conventional drying process; (**b**) Deformation of the microstructures during conventional drying process; (**c**) The microstructures fixed by gel.

During the drying process, the liquid evaporates gradually, the liquid between the structures remains much longer than that which is at the edges (Figure 1a). Then different capillary forces would occur, which lead to the deformation of the microstructures (Figure 1b). In the worst case the structures break off from the substrate [10,11]. However, if the liquid drying process enables this to be avoided, no pattern defects would occur (Figure 1c). The liquid level is avoided by preventing the direct phase change from liquid to gas in the whole process.

### 2.2. New Method

Gelation refers the process of sol forming a gel under certain conditions. Non-permanent physical gel means that the gel is formed by physical force, such as electrostatic interaction, hydrogen bonding, chain entanglement, etc. This kind of gel is able to be transformed into a sol by being dissolved in water under a certain temperature, and then converted to a gel upon cooling [12,13]. Thus, the process enables to be reversible and recycle.

**Figure 2:**
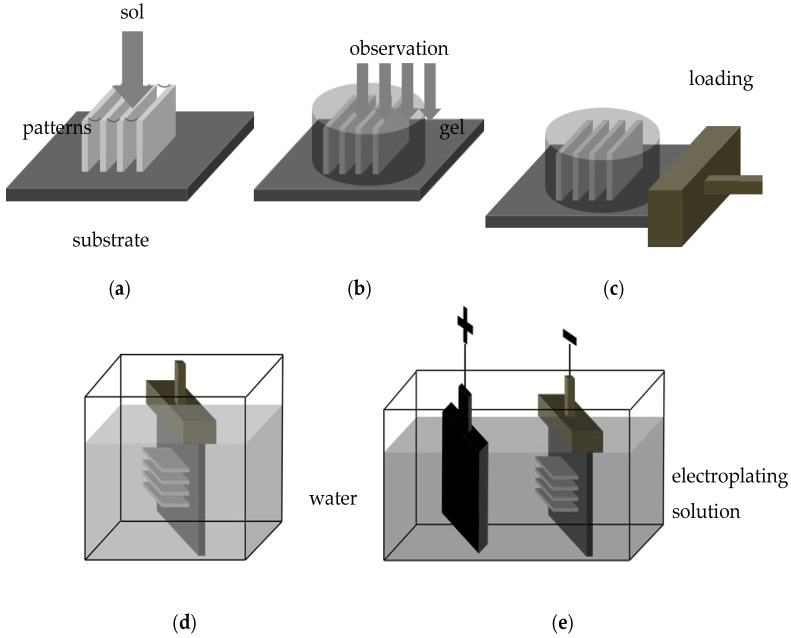
The processes of fabricating metal microstructures with the new method: (**a**) Adding the sol and forming gel; (**b**) Examination of fixing photoresist microstructures under the microscope; (**c**) Loading the sample on the holder; (**d**) Dissolving the gel in the warm water; (**e**) Electroplating process.

The new method is based on such a thermo-reversible gel. Figure 2 shows the processes of fabricating metal high-aspect-ratio microstructures with the new method. First, before the rinse liquid drying process, the sol is added to the high-aspect-ratio microstructures (Figure 2a), and it would fill the interspace between the structures. By changing the temperature, sol transforms into gel so that the structures are fixed to prevent collapsing. Then, the sample with fixed patterns can be observed (Figure 2b). After observation process, special electroplating holder would be adopted to load the sample. It is effective to form a standard electroplating process (Figure 2c). Then the sample would be immersed into the warm water for a few seconds, the gel that covered the structures can be dissolved in water again (Figure 2d). Finally, the sample is immersed into plating solution for electroplating (Figure 2e), metal high-aspect-ratio microstructures enable to be fabricated. During all these processes, no capillary forces occur, which ensures the structures are well-preserved.

The new method, freeze-drying and supercritical drying all avoid capillary forces from collapsing or damaging the high-aspect-ratio microstructures by preventing the direct phase changing from liquid to gas [14,15,16]. Freeze-drying and supercritical drying are the final step, while our new method is temporarily fixing wetted microstructures via gelation. Combined with the follow-up process to fabrication high-aspect-ratio microstructures and devices, our method fulfills the concept of “microfabrication always in liquid”. This is a facile, easily controllable and cost-effective approach. So it is more compatible with the electroplating process and wet etching process.

### 2.3. Experimental Setup

In our experiment, a series of high-aspect-ratio microstructures were fabricated by the standard lithography processes. First, a 240 μm layer of SU-8 2100 photoresist (MicroChem Corp., Westborough, MA, USA) was spin-coated on cleaned silicon wafer. Second, a two-step soft bake (65 °C for 7 min and 95 °C for 55 min) was performed on a level hot plate. Then, the sample was exposed with a series of grating dimensions using UV exposure (i-line, 360 mJ/cm^2^). After a post-exposure bake (65 °C for 5 min and 95 °C for 18 min) and sufficient cooling process, the development was carried out in the SU-8 developer for 18 min and rinsed in isopropyl alcohol (IPA) for 30 s. Then the sample was immediately kept in the deionized water.

In order to immobilize the microstructures and avoid liquid drying, the gellan gum (Macklin, Shanghai, China) sol (gellan gum: 0.6 g, water: 100 mL, 65 °C) adding process must be done as soon as possible. Then the sol was spin-coated on the sample. The recommended coating program included two parts: (1) spin at 1000 rpm for 10 s with acceleration of 100 rpm; (2) spin at 1800 rpm for 20 s with acceleration of 300 rpm. After coating process, the sample was placed on a horizontal plate until all the microstructures were fixed by gel completely. Then stereoscopic microscope (Leica Microsystems Ltd., Shanghai, China) and optical microscopy (Nikon Optiphot 100, Nikon Instruments, Co., Ltd., Shanghai, China) was used for observing the patterns. After observation process, in order to remove the gel, the sample was immersed in warm water (60 °C for 30 s).

## 3. Results and Discussion

**Figure 3:**
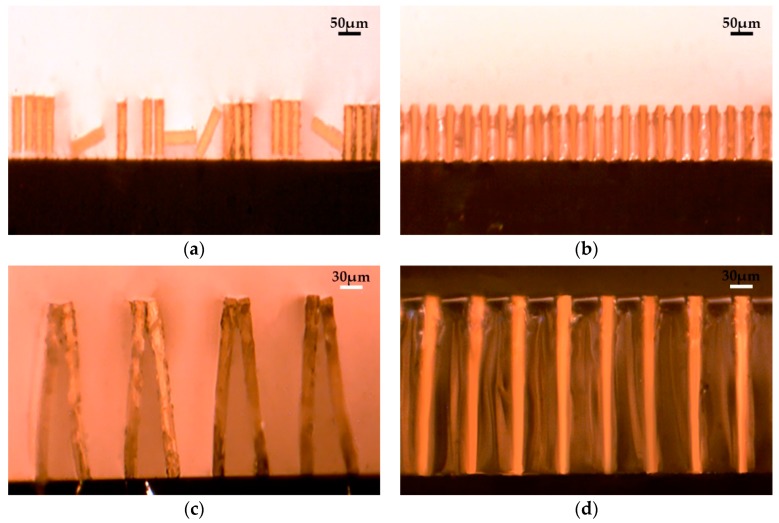
The stereoscopic microscopy images of the gratings (side views): (**a**) After the conventional drying process, some structures deformed or adhered together (width: 24 μm; height: 240 μm); (**b**) The structures were fixed by gellan gum gel (width: 24 μm; height: 240 μm); (**c**) After the liquid drying process, the top of the structures adhered to each other (width: 15 μm; height: 240 μm); (**d**) The structures were fixed by gellan gum gel (width: 15 μm; height: 240 μm).

In order to validate the new method, different high aspect ratios of microstructures were fabricated and fixed by gel. Figure 3 shows the microscopic images of two different high-aspect-ratio microstructures. Beams with an aspect ratio of 10 are shown in Figure 3a,b. After the sample was taken out of the liquid, capillary forces would be generated while the rinse liquid evaporated in the atmosphere. Then, pattern defects would occur. Different defects such as deformation, peeling off from the substrate and adhesion were generated during the drying process (Figure 3a). Fixed by gel, the beams were kept upright (Figure 3b). Beams with a higher aspect ratio of 16 are shown in Figure 3c,d. Without any treatment, the microstructures in Figure 3c adhered to each other on the top. Figure 3d shows beams were kept upright by the gel. The grooves between the structures were covered with gel, and no liquid level would occur, so the capillary forces would not be generated. Even though the thickness of the sol had not been controlled well, or some water of the gel evaporated during the observation process, the structures would still stand upright (Figure 3b). Therefore, it can be concluded that the new method is feasible and practical for fixing the microstructures. As shown in Figure 3b,d, even though the sample is covered with gel, the patterns are very clear in the observation. It can be concluded that the gel could easily be removed under certain conditions and would not cause any damage to the structures.

### 3.1. Influence of Different Kinds of Gel

As the fixed patterns need to be clear and stable for observation, it is necessary to choose a colorless and transparent gel, such as carrageenan, gellan gum, or ager. In our experiment, agarose (Sangon Biotech, Shanghai, China) and gellan gum (Macklin, Shanghai, China) sol were added separately on two samples with the same layout to show the different impacts on the microstructures. The operation process was described previously. Two kinds of sol are made as follows: agarose sol: 1% (agarose: 1.0 g, water: 100 mL), 95 °C; gellan gum sol: 0.6% (gellan gum: 0.6 g, water: 100 mL), 65 °C; ambient temperature: 25 °C.

**Figure 4:**
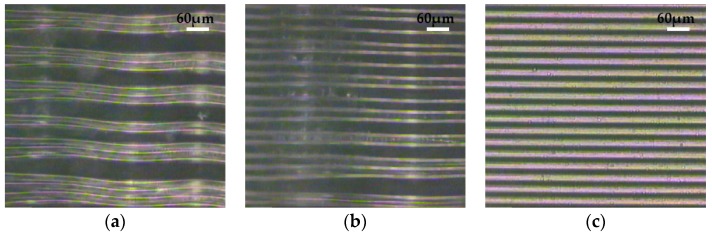
Images of SU-8 beams (width: 20 μm; height: 240 μm). (**a**) Traditional drying process; (**b**) Addition of agarose sol before the drying process; (**c**) Addition of gellan gum sol before the drying process.

After the rinsing liquid drying process, pattern defects would always occur (Figure 4a). Compared to Figure 4b, the patterns of Figure 4c were fixed in a better condition. It is obvious that gellan gum is more effective for fixing wetted microstructures than agarose, since the gellan gum sol is easier to evenly fill into gaps among the high-aspect-ratio microstructures than the agarose solution. Meanwhile, the gellan gum gel has better light transmittance. Consequently, gellan gum sol is more suitable for temporarily fixing wetted high-aspect-ratio photoresist microstructures. In consideration of the compatibility with the following electroplating process, the temperature of the gel transforming back into sol should be consistent with that of the electroplating solution. Therefore, gellan gum is a good choice to fix the microstructures.

### 3.2. Two Major Factors during the Observation Process

Gel time and observation time are two major factors during the observation process. Gel time is a period of time where sol is added to the sample and transformed into cured gel. In general, less gel time would ensure the whole process is more effective. However, an excessively short gel time will make it difficult to control the flatness and thickness of the gel layer. Observation time is a period of time that the temporarily fixed wetted microstructures could be excellently observed and examined under the microscope. The observation time starts at the formation of the cured gel and expires with the structural changes of the gel layer due to dehydration. The observation time should be sufficient in order to have enough time to perform various operations during the observation process. Both the gel time and observation time have a significant relationship with the concentrations of the sol. Therefore, to acquire considerable observation time and appropriate gel time, it is essential to sort out the sol-gel solution and the optimal concentration.

**Figure 5:**
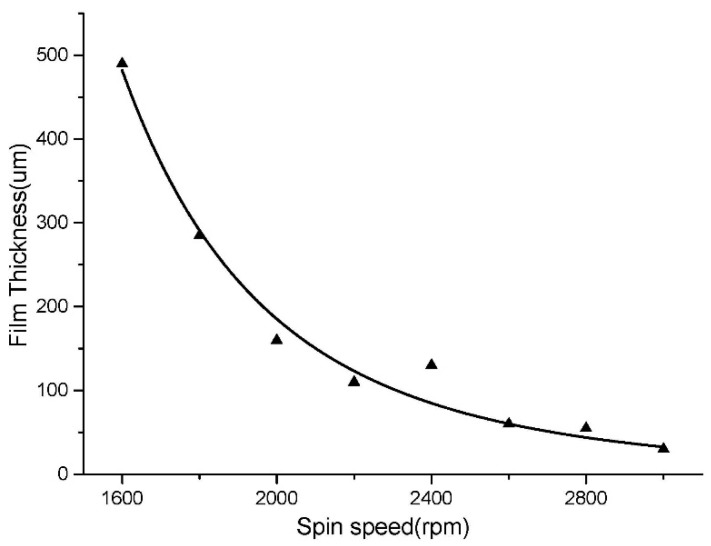
Gellan gum spin speed versus thickness.

In our experiment, spin-coating was used to control the thickness and flatness of the gellan gum gel layer. Before the spin-coating process, the sample was placed in a horizontal position for 30 s after the sol was added. The gel film thickness versus spin speed for the gellan gum sol (0.60%) is shown in Figure 5. According to Figure 5, the desired film thickness (about 300 μm) could be achieved, which is a little more than the height of the structures (240 μm). After the coating process, the sample was placed on a plate in a horizontal position for 120 s so that the sol transformed into gel completely. For different concentrations and different kinds of gel, the corresponding curve graph of the spin speed versus thickness should be measured.

**Figure 6:**
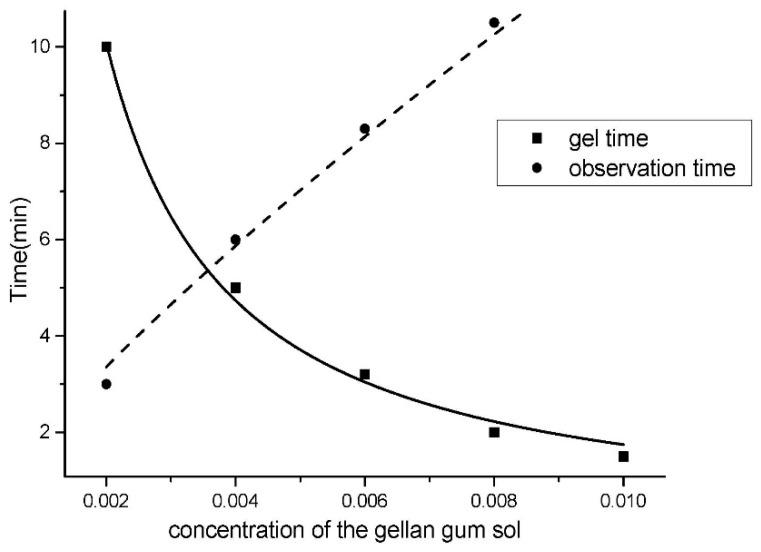
The gel time and observation time versus the concentration of the gellan gum sol.

As shown in Figure 6, the gel time and observation time of gellan gum solution under different concentrations (0.20%–1.00%) are measured. The solid line in Figure 6 indicates that with the concentration rising, the gel time of gellan gum decreases. It should be noted that further process have to wait for sol to transform into gel completely. Otherwise, moving the sample would lead to damage of the structures.The dotted line in Figure 6 indicates that the bigger the concentration of the solution, the more observation time can be obtained. At room temperature, the water-holding capacity is related to the concentration of the gellan gum sol. The higher the concentration, the better the water-holding capacity is. The observation time can be extended when the microstructures with gel are placed in a humidity chamber. Gellan gum sol with a concentration of less than 0.6% would offer less observation time. In order to obtain the same thickness, the spin speed needed to be relatively low. A low spin speed would lead to an unstable spin-coating process. It is proved that gellan gum sol with a concentration of 0.6% is relatively suitable in our experiment.

**Figure 7:**
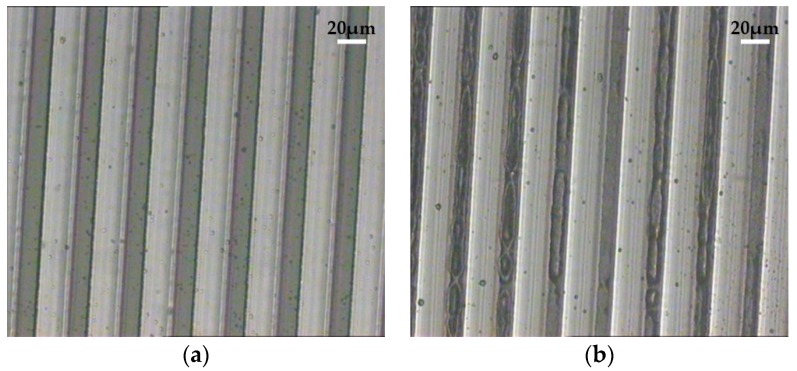
The optical microscopy images of the micro-gratings (width: 20 μm; height: 240 μm): (**a**) Within observation time; (**b**) 10 min later.

Observation time is one of the important factors that needs to be considered during the observation process. Figure 7 shows the photos of structures fixed by gellan gum gel (sol concentration: 0.60%) in two different observation periods. As shown in Figure 7a, the structures fixed by gel are able to be observed clearly. However, as shown in Figure 7b, a period of time (about 10 min) later, it is obvious that the shriveled gel deteriorated the optical properties of the sample, and even though some water in the gel evaporated in the air, the structures were still fixed upright by the gel. Thus, the observation process needs be done according to the observation time in Figure 6. In order to ensure the observation process is normative and effective, it is necessary to have good control of the gel time and observation time.

## 4. Conclusions

We proposed a new technique for temporarily fixing wetted high-aspect-ratio microstructures via gelation for the purposes of ex situ observation during the intermediate steps of wet processing. The gel is formed between wetted microstructures by the addition of a sol, preventing capillary forces from collapsing or damaging the delicate structures. Preliminary results show that microstructures with an aspect ratio of 16 were fixed by gellan gum gel. A clear and stable examination process could be achieved. Since the gel can easily be removed in warm water, the new method could be repeated and the gel recycled. With an appropriate gel, the experimental setup and procedure could be employed in other processes. Therefore, the process concept of “plating always in liquid” goes on smoothly and effectively, and the efficiency of the microstructure fabrication process could be improved greatly. In the future, the new method has the potential to be used in fabricating high-aspect -ratio nanostructures.
